# Increasing Ebola transmission behaviors 6 months post-vaccination: Comparing vaccinated and unvaccinated populations near 2018 Mbandaka Ebola outbreak in the Democratic Republic of Congo

**DOI:** 10.1016/j.vaccine.2021.10.071

**Published:** 2021-12-17

**Authors:** Nicole A. Hoff, Anna Bratcher, Patrick Mukadi, Steve Ahuka, Michel Kabamba, Kamy Musene, Megan Halbrook, Camille Dzogong, Guillaume Ngoie Mwamba, Placide Mbala, J. Daniel Kelly, Jean Paul Kompany, Merly Tambu, Didine Kaba, Benoit Kebela-Ilunga, Jean Jacque Muyemebe-Tamfum, Anne W. Rimoin

**Affiliations:** a Department of Epidemiology, University of California, Los Angeles, Fielding School of Public Health, Los Angeles, CA, USA; b Institut National de Recherche Biomédicale, Kinshasa, Congo; c National Expanded Program for Immunization, Kinshasa, Congo; d UCLA-DRC, Kinshasa, Congo; e School of Medicine, University of California, San Francisco, San Francisco, CA, USA; f Ecole de Sante Publique, Université de Kinshasa, Kinshasa, Congo; g Direction de lutte contre la Maladie-Ministère de la Santé Publique, Kinshasa, Congo

**Keywords:** Ebola, Vaccination behaviors, rVSV-EBOV, Democratic Republic of the Congo (DRC), Post-outbreak behaviors, Post-vaccination behaviors

## Abstract

**Background:**

In 2018, the Democratic Republic of the Congo (DRC) declared its 9th and 10th Zaire ebolavirus (EBOV) outbreaks, in the Equateur province (end: July 2018), and in the eastern provinces including North Kivu (end: June 2020). The DRC Ministry of Health deployed the rVSV-vectored glycoprotein (VSV-EBOV) vaccine in response during both outbreaks.

**Methods:**

A cohort of vaccinated and unvaccinated individuals from the Equateur province were enrolled and followed prospectively for 6 months. Among participants included in this analysis, 505 were vaccinated and 1,418 were unvaccinated. Differences in transmission behaviors pre- and post- outbreak were identified, along with associations between behaviors and vaccination.

**Results:**

There was an overall increase in the proportion of both unvaccinated and vaccinated individuals in Mbandaka who participated in risky activities post-outbreak. Travel outside of the province pre-outbreak was associated with vaccination. Post-outbreak, vaccinated individuals were less likely to participate in funeral traditions than unvaccinated individuals.

**Conclusion:**

A net increase in activities considered high risk was observed in both groups despite significant efforts to inform the population of risky behaviors. The absence of a reduction in transmission behavior post-outbreak should be considered for improving future behavior change campaigns in order to prevent recurrent outbreaks.

## 1 Introduction

In 2018, the Democratic Republic of the Congo (DRC) declared its 9th and 10th Zaire ebolavirus (EBOV) outbreaks [1]. The first of which occurred in the western province of Equateur and was declared over in July 2018 [2]. Meanwhile, the second outbreak, starting in August 2019 lasted almost 2 years, only declared over on June 25, 2020 [3]. As a part of the response efforts for both outbreaks, the DRC Ministry of Health deployed a compassionate use protocol for the use of the rVSV-vectored glycoprotein (rVSV-EBOV) vaccine manufactured by Merck & Co. which has since been licensed for use by the FDA. [4]

While this vaccine has shown promising results in preliminary trials [5], [6], we still do not have a thorough understanding of how this vaccine impacts EBOV outbreaks response and post-outbreak. Of particular importance is how this vaccine may affect risk behaviors such as performing funeral rites or handling dead animals, both during an outbreak or after EVD cases are no longer occurring in an area. While behavior changes following EBOV vaccination are currently unstudied, research with transmission behaviors following other vaccinations demonstrate that this is plausible. For example, it has been shown that there is generally a decrease in HPV risk behaviors after being vaccinated for HPV [7].

Understanding changes in risk behavior in vaccinated and unvaccinated individuals is a crucial piece to knowing how the deployment of a vaccine could impact future outbreaks, which is not only important in the context of EVD outbreaks but others such as the current worldwide pandemic of COVID-19. Regardless of vaccine efficacy, unvaccinated individuals are subject to elevated risk according to their behavior patterns. Vaccinated individuals are much less likely to experience infection, but still retain some level of post-vaccination EVD risk [5], [6], [8], [9]. Additionally, if future studies find that immunity wanes over time or there is reduced vaccine effectiveness in certain settings, such as low resource areas where maintaining cold chain is a challenge [10], [11], it is important to understand if vaccinated individuals experience elevated risk due to their behaviors based on perceived protection.

In addition to the vaccination’s potential effect on behavior, it is also important to understand how behavior changes following the final cases of an EVD outbreak. Given new evidence of viral persistence in human survivors [12], it is important to understand if risky behavior rebounds as outbreaks end. While the risk of exposure may be lower after the official end of an outbreak, these communities are still at risk for recurrent outbreaks where index cases may or may not be vaccinated [1]. Therefore, we must know if these communities tend to have an increase in high-risk behavior following the final cases of an outbreak. If so, efforts must be made to change this pattern.

The 2018 EBOV outbreak in Mbandaka, DRC provides an opportunity to observe behavior change in an area with both vaccinated and unvaccinated individuals before and after an EBOV outbreak. This paper uses data collected from vaccinated and unvaccinated populations surrounding the Mbandaka EBOV outbreak to examine how behavior changes from prior to an EBOV outbreak to the 6-month period following EVD cases in the area. Changes in behavior could inform how members of Ebola affected communities respond to the end of outbreaks and contribute to our understanding of the risk of recurrent outbreaks. Additionally, we will compare how changes in behavior varied by vaccination status in our sample, both prior to and following EVD cases in the area. As vaccination for Ebola becomes more common, it is crucial to understand how this affects risk behavior.

## 2 Methods

As a part of a larger study to explore the humoral immune responses and durability of these responses in participants post vaccination, cohorts of Merck & Co. rVSV ZEBOV-GP vaccine recipients along with unvaccinated individuals in Mbandaka city, made up of three health zones (Wangata, Mbandaka and Bolenge) were enrolled between June and July 2018 and followed prospectively. Participants were recruited after the final case of the 2018 EVD Mbandaka was confirmed, but before the end of the outbreak was officially declared on July 24th, 2018 (42 days after the last confirmed case).

Briefly, vaccinated participants were offered enrollment into the cohort if they were vaccinated as a part of the ring vaccination strategy implemented by the Expanded Programme for Immunization (EPI) and WHO. This strategy focused on setting up vaccination sites in locations near confirmed cases and targeted contacts of confirmed EBOV cases, contacts of contacts and first responders/health care workers. In order to ensure vaccination activities took priority, participants were not approached for enrollment until after their vaccination and observation for adverse events was complete and they were leaving the vaccination site. This method helped ensure all consenting vaccinated participants had received vaccination. The unvaccinated cohort in Mbandaka included healthcare workers, close contacts of Ebola patients, and others from the general population collected through convenience snowball sampling. This method included randomly selecting health facilities throughout the three health zones and offering enrollment to all health care workers (including traditional healers and pastors). After enrollment of the primary groups (vaccinated individuals and unvaccinated health care workers, each participant was provided with an invitation to refer one additional person to participate as a member of the general unvaccinated population.

Questionnaires and blood samples were collected from consenting participants at a vaccination visit (or a baseline visit for unvaccinated participants) and at multiple follow-up visits. Informed consent was required for involvement in the study and participants had the right to refuse participation at any time. This analysis uses questionnaire data from the vaccination/baseline visit and the 6-month post-outbreak follow-up. Questionnaires were conducted by trained interviewers in the local language (French or Lingala), and collected data on demographics, potential exposures to Ebola virus, transmission behaviors for Ebola virus, animal exposures, and occupational exposures. To assess transmission behavior, participants were asked if they had done any of the following activities in the 6 months prior to the outbreak or prior to the 6 month follow up: attended a funeral, had direct exposure to human remains, participated in funeral traditions, came in contact with dead animals, traveled outside of locality (province), frequented markets, or visited a health facility for an ailment. This list represents the closest English translation from the local languages used in the survey.

A descriptive statistical analysis was conducted on sample characteristics as well as on behavioral variables. Demographic descriptors were obtained for the vaccinated and unvaccinated groups. The percent of each group that participated in each behavior in the 6 months prior to the outbreak and in the 6 months between the baseline and 6-month follow up visit were calculated. A 95% confidence interval for percent change over time was obtained using generalized estimating equations accounting for the paired nature of the data. All statistical analyses were carried out using SAS software, version 9.4 (SAS Institute, Cary, NC).

**Fig. 1:**
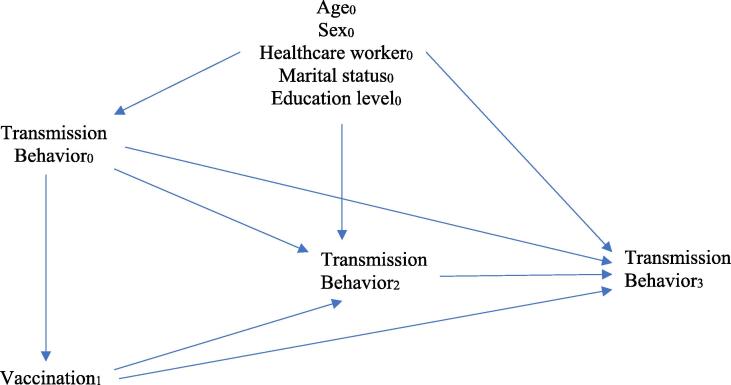
Directed Acyclic Graph (DAG) used to determine variables needed for bias correction in models.

Adjusted odds ratios for transmission behaviors comparing vaccinated individuals to unvaccinated individuals at both time points were obtained using generalized mixed linear models. These odds ratios were adjusted for age, sex, marital status, healthcare worker status, and education according to a priori indication as confounders according to the hypothesized Directed Acyclic Graph (DAG) (Fig. 1).

This study was approved by Institutional Review Boards (IRBs) at the University of Kinshasa in Kinshasa, DRC (ESP/CE/022/2017) and at the University of California, Los Angeles (UCLA). Additionally, the study was approved by the Scientific Committee for Ebola Research during an outbreak at the National Institute of Biomedical Research (INRB) under the Ministry of Health. Before any study-related procedures were conducted, participants signed or marked the approved informed consent form.

## 3 Results

Five hundred and five (505) vaccinated participants in Mbandaka were recruited, 422 (84%) of which had behavioral data at the 6 months post-vaccination visit. Of the 1,418 unvaccinated individuals recruited, 1,166 (82%) had behavioral data at 6 months of follow up.

Our vaccinated and unvaccinated samples significantly differed in distributions of age, sex, education, marital status, tribe, and occupation across vaccination status (Table 1). The vaccinated population was older and more educated than the unvaccinated population. Additionally, vaccinated individuals were more commonly male and married or living with a partner as married.

**Table 1:** Sample characteristics of vaccinated and unvaccinated individuals from Mbandaka, Democratic Republic of the Congo 2018.

	**Vaccinated**		**Unvaccinated**		**Comparison**	
	**n = 505**		**n = 1418**			
	Mean	Standard Deviation	Mean	Standard Deviation	Mean difference	p-Value
Age	41	12.45	37	13.55	4	<0.0001

	Count (n)	Percent (%)	Count (n)	Percent (%)	chi-square	p-Value

Sex						
*Male*	352	69.7	807	56.9	25.45	<0.0001
*Female*	153	30.3	611	43.1		
Age						
*18*–*24*	47	9.3	314	22.1	71.02	<0.0001
*25*–*34*	121	24.0	431	30.4		
*35*–*44*	162	32.1	284	20.0		
*45*–*54*	110	21.8	217	15.3		
*55*–*64*	45	8.9	125	8.8		
*65*–*85*	20	4.0	47	3.3		
Education^a^						
*None or some primary school*	12	2.4	80	5.6	107.30	<0.0001
*Finished primary school or apprenticeship*	57	11.4	375	26.4		
*Finished secondary school*	151	30.1	512	36.1		
*College/University or Graduate school*	282	56.2	451	31.8		
Marital status^b^						
*Single*	117	23.3	490	34.6	36.41	<0.0001
*Married or living together as married*	368	73.2	825	58.2		
*Divorced, separated, or widowed*	18	3.6	103	7.3		
Religion^a^					10.39	0.0343
*Catholic*	148	29.5	500	35.3		
*Protestant*	120	23.9	278	19.6		
*Eglise de reveil*	171	34.1	498	35.1		
*Muslim*	10	2.0	28	2.0		
*Other*	54	10.8	113	8.0		
Tribe						
*Ekonda*	42	8.3	141	9.9	21.90	<0.0001
*Mongo*	175	34.7	617	43.5		
*Ngombe*	58	11.5	178	12.6		
*Other*	230	45.5	482	34.0		
Occupation^c^						
*Farmer, fisher, or hunter*	59	11.8	260	18.4	123.00	<0.0001
*Teacher*	17	3.4	52	3.7		
*Healthcare worker*	187	37.3	543	38.3		
*Merchant*	22	4.4	155	10.9		
*Technician*	11	2.2	18	1.3		
*Student*	39	7.8	173	12.2		
*Driver*	13	2.6	35	2.5		
*Politics*	4	0.8	26	1.8		
*Other*	150	29.9	154	10.9		

a Missing 3 from vaccinated group.

b Missing 2 from vaccinated group.

c Missing 3 from vaccinated group; missing 2 from unvaccinated group.

From the 6 months preceding the outbreak to the 6-month follow-up, there was an overall net increase in the proportion of vaccinated individuals who participated in the following activities that may increase EBOV risk: funeral attendance, direct contact with human remains and dead animals, and participation in funeral rights (Table 2). For unvaccinated individuals, there were similar increases in these behaviors, except for contact with dead animals. The largest increase in risk behavior post outbreak was an 17.3% increase in those who participated in funeral traditions in the vaccinated population.

**Table 2:** Percent of sample that participated in Ebola transmission behaviors and crude change in percent participation between 6 months prior to the 2018 EVD outbreak and 6 months post-EVD cases in Mbandaka, Democratic Republic of Congo 2018–2019.

	Vaccinated			Unvaccinated		
	6 months prior to outbreak declaration n = 505	Between baseline visit and 6 month follow up n = 422		6 months prior to outbreak declaration n = 1418	Between baseline visit and 6 month follow up n = 1166	
	Percent (%)	Percent (%)	Percent Change (95% CI)	Percent (%)	Percent (%)	Percent Change (95% CI)
**Activity performed in prior 6 months**						
Attended funeral	33.9	48.3	14.4 (8.8, 20.1)	39.8	49.1	9.3 (5.8, 12.9)
Had direct exposure to human remains	21.5	34.1	12.6 (6.9, 18.3)	21.3	32.4	11.1 (7.7, 14.5)
Participated in funeral traditions	35.8	53.1	17.3 (11.1, 23.5)	42.8	54.7	11.9 (8.2, 15.7)
Came in contact with dead animals	6.3	11.0	4.6 (1.1, 8.2)	7.3	9.3	2.1 (0, 4.2)
Traveled outside of locality (province)	34.7	37.7	3.0 (−2.3, 8.3)	28.8	26.0	−2.8 (−6.0, 0.3)
Frequented markets	86.1	87.9	1.8 (−2.1, 5.7)	89.9	89.4	−0.5 (−2.91.8)
Visited health facility for an ailment	41.4	40.6	−0.8 (−6.7, 5.1)	40.1	40.1	0 (−3.5, 3.5)

CI – Confidence Interval.

Only two behaviors showed evidence of an association with vaccination status (Table 3). Those who were vaccinated were more likely to travel outside of the province pre-outbreak (OR 1.78, 95% CI 1.33, 2.38), holding confounders constant. In the 6 months of follow up, vaccinated individuals were less likely to participate in funeral traditions than unvaccinated individuals (OR 0.78, 95% CI 0.62, 0.97), holding confounders constant.

**Table 3:** Adjusted odds ratios for performing Ebola transmission behaviors in the 6 months prior to the 2018 EVD outbreak and 6 months post-EVD cases among vaccinated individuals compared to unvaccinated individuals in Mbandaka, Democratic Republic of Congo 2018–2019.

	6 months prior to outbreak			Between baseline visit and 6 months follow up		
	Odds Ratio*	95% Confidence Interval	p-Value	Odds Ratio*	95% Confidence Interval	p-Value
**Behavioral outcomes**						
Attended funeral	1.04	0.80, 1.5	0.7651	0.80	0.63, 1.03	0.0796
Had direct exposure to human remains	1.24	0.97, 1.59	0.0860	1.21	0.93, 1.57	0.1485
Participated in funeral traditions	1.03	0.81, 1.30	0.8336	0.78	0.62, 0.97	0.0276
Came in contact with dead animals	1.44	0.67, 3.13	0.3537	0.66	0.26, 1.68	0.3823
Traveled outside of locality (province)	1.78	1.33, 2.38	<0.0001	1.27	0.97, 1.65	0.0834
Frequented markets	1.16	0.76, 1.77	0.4825	0.91	0.62, 1.33	0.6327
Visited health facility for an ailment	0.93	0.72, 1.20	0.5522	0.94	0.75, 1.20	0.6315

* Controlled for age, sex, marital status, healthcare worker status, and education.

## 4 Discussion

Both vaccinated and unvaccinated cohorts surrounding the 2018 Mbandaka EBOV outbreak showed a net increase in activities considered high risk for Ebola such as attending funerals, participating in funeral rights, and touching human remains. These increases in behavior ranged from a 9.3% net increase in attending a funeral in the unvaccinated sample, to an 17.3% net increase in participation in funeral traditions among vaccinated individuals. These changes were observed despite significant efforts to inform the population of risky behaviors [13]. If high risk activities continued to increase or remained at high levels for an extended period, this may have been a contributing factor to the recent EBOV outbreak declared in Mbandaka in June 2020, a year and a half after the 6 month follow up visit in this analysis.

In addition to the overall participation in behaviors prior to and following EVD cases in the area, our study also allowed us to assess how behavior change may vary across vaccination status. Our study suggests that vaccinated individuals were more likely to travel outside of the province prior to the outbreak compared to the 6 month follow up period. In contrast, vaccinated individuals were less likely to participate in funeral traditions in the 6 months following EVD cases in the area. There are multiple reasons we may have observed this, this could be an indication that certain parts of the sensitization for reducing risky behavior may have influenced change in behaviors. It could also be linked to vaccinated persons in general overall healthier and being surrounded by other vaccinated people, and less likely to have those close to them die. Further exploration is needed to understand possible causal mechanisms behind the observed association.

While our findings are similar to other postvaccination behavior studies, mostly regarding HPV vaccination [7], a number of limitations exist. Data was collected initially during an active outbreak period, which may have been a more stressful period. This stress may have impacted those who participated in the study. Additionally, data was collected through convenience sampling methods and responses on exposures were self-reported, which may be subject to bias due to sampling, limitations of recall, and translation errors. Recall bias could have made post-outbreak behavior more salient to participants, resulting in the observed increases in risk behaviors for the follow up period compared to the pre-outbreak period. While there was little to be done about limitations of recall, it is likely these would be similar between the two cohorts and much effort was undertaken to reduce information bias due to translation errors. Local staff were hired to administer questionnaires in order to conserve information in each translation from local languages to English and vice versa. Furthermore, it is possible that our selected covariates did not completely eliminate confounding in our Table 3 estimates. There may have been residual confounding from sources that were unmeasured in this cohort, such as wealth or income. Unfortunately, though sexual intercourse has been noted as a potential risk in recovered persons and may be linked to emergence to current outbreaks, this was not discussed during our survey. One strength of this study is the high follow up rate (>80% in both vaccinated and unvaccinated individuals 6 months post initial enrollment), despite difficult conditions. This is a reflection of the strong local study staff and on the ground knowledge of working with mobile populations as well as strong community knowledge of the study activities.

Ultimately, this study contributes to our understanding of how behaviors may change in vaccinated and unvaccinated individuals following Ebola outbreaks. Our data suggests that many high-risk behaviors either do not change or increase following the final cases of an EVD outbreak. This may be indicative of the perceived risk of these activities and how sensitization activities informed the population of the risk for both vaccinated and unvaccinated individuals. More research is needed to determine if this increase in risk behaviors in general may have contributed to Mbandaka’s recurrent outbreak declared in June 2020. In addition, these results indicate the potential absence of a permanent reduction of risk behaviors and should be taken into consideration in Ebola outbreak response strategies to motivate long term reduction in high risk behaviors. Further, these results may be indicative of a general need for improved messaging and sensitization during outbreaks, which is especially important given the current COVID-19 worldwide pandemic, which has already had a devastating effect on much of the world's population yet, many still participate in high risk activities for continuing transmission.

## Declaration of Competing Interest

The authors declare that they have no known competing financial interests or personal relationships that could have appeared to influence the work reported in this paper.
